# Hypophospite of Zinc

**Published:** 1878-05-20

**Authors:** R. W. Gardner


### HYPOPHOSPHITE OF ZINC.

SIR: This salt has never, so far as I can learn, been used internally for medicinal purposes, and the object of this paper is to explain its peculiar advantages and to call professional attention to it.

The only combination of phosphorus and zinc heretofore used is the phosphide of zinc. The formula for this, as given in Soubeiran's work as recently revised by Regnault, is $PZn_3$. This shows it to consist of phosphorus and metalic zinc in the proportion of one part of the first to about six parts of the latter, and both unoxidized. As no oxygen enters into its combination, the phosphorus produces the same irritant effect in the stomach as if given in the free state, its irritant action being caused by its oxidation.

The ordinary dose of phosphide of sinc is one-tenth grain. This quantity cannot be largely increased. The proportion of zinc contained in this quantity is too infinitesimal to prove a very powerful nervous tonic, while the proportion of phosphorus is still less.

I therefore propose the use of hypophosphite of zinc. The formula for this is (ZnO, 6HO, PO) O=8).

In this salt both the zinc and phosphorus are in a protoxidized state, in which condition the phosphorus is rendered non-irritant, admitting the use of such quantities as to fully meet all indications for either phosphorus to zinc. The salt being perfectly soluble, is at once assimilated, while both elements in the phosphide must be oxidized previous to assimilation. Its advantage in most nervous diseases over zinc oxide, zinc sulphate, etc., being that the very desirable effects of phosphorus in its best condition for assimilative action are also available by its use.

The condition of absolute purity is essential to this, as to all hypophosphite salts, for unless used in this condition their therapeutic effect is either very much impaired or wholly lost.

I recommend the use of the syrup, as the sugar is a most efficient preserver against atmospheric influence, and renders the preparation more palatable, though the salt has scarcely any taste, at first, a slight metalic after-taste being perceptible.

It is prepared in the form of syrup, eight grains of the salt to one fluid ounce, and may probably be given in twice or thrice that quantity three times a day.

The salt used is purified and neutral. It has been therapeutieally tested with very satisfactory results.—R. W. GARDNER, in *New York Med. Rec.*

